# The use of molecular cues to regenerate musculoskeletal tissues

**DOI:** 10.1515/iss-2025-0033

**Published:** 2025-09-24

**Authors:** Carlos Julio Peniche Silva, Martijn van Griensven, Virginie Joris

**Affiliations:** Cell Biology-Inspired Tissue Engineering, 534871 MERLN Institute for Technology-Inspired Regenerative Medicine, Maastricht University, Maastricht, The Netherlands

**Keywords:** musculoskeletal, molecular cues, repair, regeneration, healing

## Abstract

**Introduction:**

Musculoskeletal tissues, including bone, tendon, cartilage, and muscle, are vital for movement and structural support, yet, their repair after injury remains a significant clinical challenge. Their regeneration relies on complex molecular signaling that regulates inflammation, repair, and remodeling. Understanding these cues, offers opportunities to design targeted therapeutic strategies.

**Content:**

This review summarizes current evidence on molecular regulators of musculoskeletal tissue regeneration with emphasis on both shared and tissue-specific mechanisms across bone, tendon, cartilage and muscle. Key molecular cues include growth factors, cytokines, extracellular matrix-derived signals, and non-coding RNAs, particularly microRNAs. Critical pathways such as TGF-β, NF-κB, FGF, and YAP/TAZ can either promote healing or drive pathological fibrosis depending on their modulation. This review discusses therapeutic strategies targeting these molecular cues, including microRNA replacement therapies, small molecules, growth factor delivery, and pathway-specific inhibitors or activators.

**Summary and Outlook:**

Understanding how these molecular cues and pathways function and interact to regulate healing and regeneration offers valuable insight into tissue-specific and cross-tissue repair strategies. These advances may support the development of targeted therapies to enhance musculoskeletal regeneration and functional recovery. Furthermore, future research should focus on integrating these molecular insights with biomaterial and mechanobiological approaches to develop next-generation regenerative interventions.

## Introduction

The musculoskeletal system is a complex, interconnected network of bones, tendons, muscles, cartilage, and other connective tissues that work together to give the body structure, absorb mechanical load, and enable movement [1]. Bones are the hard, mineralized connective tissue that provide structural support for the body, serving as anchoring sites for muscles and tendons, allowing locomotion, protecting soft tissues, and concealing the bone marrow [2]. Contrary to bones, muscles are soft, contractile tissues that attach to bone via tendons. Upon muscle contraction, the tendon pulls the bone, generating movement. Moreover, where two bones meet, a joint is formed. At the joints, the articular cartilage confers the necessary smooth, low-friction surface to enable bones to glide over each other during movement [3]. Each one of the highly specialized tissues that makes up the musculoskeletal system is exposed to different degrees of wear, overuse, and injuries, which can lead to different grades of disabilities, ranging from mild discomfort and pain to complete physical disability. While bones can remodel and heal with relatively high fidelity, tissues like tendons, ligaments, and cartilage have a limited regenerative capacity, often resulting in fibrosis or chronic dysfunction after injury [1], 4].

Advances in the field of tissue engineering and regenerative medicine have allowed the development of novel strategies to address musculoskeletal injuries and defects in a faster and more efficient manner than the conventional clinical approach. By combining biomaterials, cells, and signaling factors, such strategies aim to enhance and promote the regenerative capacity of the injured tissue rather than the alternative, often fibrotic healing [5], 6]. A key element of these approaches is the concept of molecular cues: biochemical and biophysical signals that orchestrate cellular behavior during tissue repair and regeneration. These cues include growth factors, cytokines, and other epigenetic regulators such as microRNAs, each influencing processes like inflammation, cell recruitment, differentiation, and matrix remodeling. This review aims to summarize the most recent advances in the use of molecular cues to address tissue repair and regeneration in the context of the musculoskeletal system.

## Search strategy

For this review, relevant literature was identified using PubMed and the Maastricht University database collection, specifically DOAJ-Directory of Open Access Journals and Europe PubMed Central, and Scopus. The search strategy included terms such as tissue regeneration, microRNA replacement therapies, small molecules, cytokines, pathway modulation, fibrosis, non-union, immunomodulation, growth factors, and RNA therapeutics, specifically concerning bone, tendon, cartilage, and muscle tissues. Priority was given to peer-reviewed articles published within the last five years; however, older publications were included when necessary to provide context or foundational insight. Conference proceedings, preprints, non-peer-reviewed articles, and case reports were excluded from the literature search.

## Bones and bone healing

Bones are stiff and strong mineralized tissue that, upon injury, undergo fracture healing via one of two mechanisms. The first, known as primary fracture healing, is characterized by intramembranous bone healing that occurs in conditions of absolute stability of the injured site, where the experienced mechanical strain falls below 2 % and yields a regenerated tissue indistinguishable from the native bone tissue [7]. Alternatively, the secondary fracture healing occurs in non-rigid fixations with a mechanical strain between 2 and 10 % [8], 9]. Secondary fracture healing occurs via endochondral healing and involves the formation of a fracture hematoma, granulation, callus formation, and bone remodeling. Furthermore, when the experienced strain at the fracture site is higher than 10 %, the fracture results in delayed union or non-union [10], 11].

To support and coordinate these healing mechanisms, bone regeneration relies on a tightly regulated network of molecular cues. Tissue engineers working on bone regeneration are constantly exploring ways to harness these cues to address the healing of challenging fractures such as those involving critical-size bone defects or complicated cases due to co-morbidities such as osteoporotic fractures.

**Figure 1:**
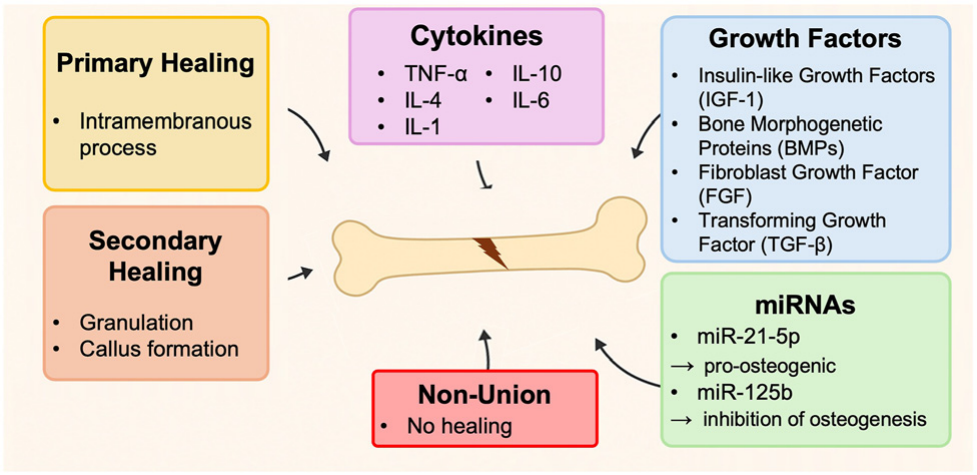
Schematic overview of bone fracture healing. Under rigid fixation (strain<2 %), primary healing restores native lamellar bone via direct osteoclast/osteoblast remodeling of haversian systems across the fracture. With micromotion (strain 2–10 %), secondary healing proceeds through a hematoma and cytokine-driven inflammation, granulation tissue and soft callus formation, angiogenesis, and hard callus bridging before remodeling to lamellar bone. Excessive strain (>10 %) causes non-union by preventing stable callus formation. Throughout repair, cytokines (early TNF-α/IL-1/6; later IL-4/IL-10) regulate inflammation and cell recruitment; growth factors coordinate matrix synthesis and vascular ingrowth; and miRNAs (e.g. miR-21-5p,miR-125 b, and others) fine-tune gene expression for optimal bone repair.

In the context of bone healing, the most often investigated growth factors are the family of bone morphogenetic proteins (BMPs), the fibroblast growth factors (FGFs), the insulin-like growth factor (IGF), and the transforming growth factor-beta (TGF-β) [12]. Additionally, cytokines released by immune cells like macrophages and neutrophils are known to be involved in the early stages of healing [13]. A balance between pro- and anti-inflammatory cytokines is crucial for efficient cellular recruitment, inflammation, and effective progression of fracture healing [14]. In these processes, the pro-inflammatory cytokines TNF-α, IL-1, and IL-6 help recruit osteoblasts and osteoclasts, while the anti-inflammatory IL-4 and IL-10 help modulate inflammation and activate osteoblasts to ensure optimal bone healing [14], 15]. Additionally, microRNAs are being extensively investigated in the context of bone healing due to their regulatory potential over gene expression and protein production [16], 17] (Figure 1).

### Molecular cues in bone tissue engineering

Several members of the BMP family are in the spotlight of tissue engineers aiming to accelerate or improve the quality of bone healing. Of these, perhaps the most studied is BMP-2, a known master regulator of bone formation. Upon interaction with its receptors (BMPRI and BMPRII), BMP-2 activates the SMAD1/5/8 pathway, regulating the expression of Runt-related transcription factor 2 (RUNX2), and Osterix [18], 19]. The osteogenic marker RUNX2 is an essential regulator of osteogenic differentiation. Furthermore, Osterix acts downstream of RUNX2 and is required for the maturation of pre-osteoblasts into functional osteoblasts [20]. BMP-2 can also activate non-Smad pathways such as the MAPK/ERK/PI3K/TAK1 pathways as well as the RhoA/Rb pathway [21], 22]. These signaling events lead to the differentiation of osteoblast precursors into osteoblasts, except for the TAB1/TAK1 pathway, which activates nuclear factor-κB (NF-κB) and inhibits osteoblast function [23], 24].

Applications of BMP-2 as a therapeutic agent have shown important actions by enhancing and promoting the formation of new bone and bridging critical-sized bone defects. Meng et al. developed a mineralized tissue-derived ECM-modified bone ceramic to deliver BMP-2 *in vivo* and used it to treat a calvarial bone defect in rodents. They reported favorable bone regeneration with enhanced alkaline phosphatase activity and expression of other osteogenic markers compared to the scaffolds alone [25]. Similarly, combining the delivery of recombinant BMP-2 with the use of biomaterials like collagen, poly(lactic-co-glicolic acid), alginate, and ceramic-based cements among others have yielded better, or at least comparable, bone healing to that of autologous bone grafting in terms of mechanical strength and bone density [26], 27]. Moreover, other members of the BMP family, such as BMP-4, BMP-7, and BMP14, among others, are recognized to be osteoinductive, although to date, only the recombinant variants of BMP-2 and BMP-7 have been approved for their clinical use in humans [28], [29], [30]. BMP-2 protein has been used in clinical situations and often off-target. Several warnings have been published. Therefore, alternatives are sought.

More recently, the use of chemically modified mRNA (cmRNA) encoding for BMP-2 has proven to offer superior bone regeneration while addressing the limitations associated with the supraphysiological application of the recombinant variants of BMP-2 to treat bone defects [31], [32], [33]. One of the most impactful studies on cmRNA-BMP-2 was published by De la Vega et al. where for the first time, it was demonstrated that cmRNA-based therapy could heal large, critical-sized segmental osseous defects in a superior fashion to its recombinant protein counterpart [33].

Furthermore, in relationship with the role of cytokines as molecular cues in bone tissue engineering, Panos et al. demonstrated that the inhibition of the IL-1-receptor using an IL-1 receptor antagonist (IL-1Ra) could yield bone healing of large, segmental bone defects in rodents utilizing doses 90 % lower of recombinant BMP-2 compared to the often-utilized supraphysiological dose. Such inhibition promoted a shift from intramembranous ossification, which is typically observed when using high doses of recombinant BMP-2, towards endochondral ossification. This resulted in better quality of healing without reduction in mechanical strength [34]. Studies such as this one exemplify the potential use of immunomodulation to potentiate the effects of growth factors at lower dosages to achieve better quality of bone healing.

Moreover, in fracture healing, the crosstalk between immune cells and mesenchymal stem cells (MSCs) drives the processes of inflammation, cell recruitment, osteogenesis, and angiogenesis [35]. Macrophages play a crucial role in this crosstalk. M1 macrophages are involved in cell recruitment and inflammation. However, M1-induced acute inflammation is resolved afterwards thanks to the polarization from M1 to M2 macrophages [36]. The absence of such polarization can lead to prolonged chronic inflammation, which consequently leads to MSC apoptosis and failed fracture healing [37]. Some studies have shown that facilitating the polarization of M1 to M2 can positively impact bone remodeling during healing. For instance, Lin et al. 2017 developed a novel preconditioning strategy for MSCs. They described that treatment with lipopolysaccharide (LPS) and tumor necrosis factor alpha (TNF-α) optimized the immunomodulatory ability of MSCs on macrophage polarization in a co-culture system. Their preconditioning strategies enhanced M2 polarization while decreasing the inflammatory M1 population, which led to increased osteogenic differentiation of the MSCs, higher alkaline phosphatase activity, and matrix mineralization [38].

Additionally, in a comprehensive review published by Frade et al. they summarized compelling evidence demonstrating that, even though most of the published work on immunomodulation and macrophages supports the notion that the right balance between M1/M2 populations is essential for managing inflammation and healthy fracture healing, the role of macrophages in fracture healing goes far beyond the regulation of inflammation [39]. Although the details on the regulatory mechanisms of macrophages during bone healing and the associated molecular cues are still to be fully elucidated, macrophages are known to recruit and activate osteoblasts, MSCs, and osteoclasts, participating in all the phases of bone healing [39], 40].

Another superfamily of molecules that has relatively recently gained significant attention is microRNAs (miRNAs). MiRNAs are naturally occurring, short non-coding sequences of RNA that can be complementary to the untranslated region (UTR) of a given mRNA sequence, targeting it for destruction, thus inhibiting protein production post-transcriptionally [41], 42]. The involvement of miRNAs in the regulation of protein expression has inspired tissue engineers to target miRNAs as therapeutic candidates. In bone tissue engineering, one of the best-studied miRNAs is miR-21–5p [43], [44], [45]. Yang et al. demonstrated that miR-21–5p has a strong pro-osteogenic, pro-angiogenic, and anti-apoptotic effect. In their study, transfections with mimics of miR-21-5p increased the level of expression of vascular endothelial growth factor (VEGF), BMP-2, Osteopontin, Osteocalcin, Runx2, and the signaling molecules Akt and Pi3K in a dose-dependent manner. Moreover, it was shown that the utilization of an MSC-laden β-TriCalciumPhosphate scaffold to treat a calvarial bone defect yielded better bone healing upon transfection with miR-21-5p [46]. A part of the osteogenic effect described for this miRNA is thought to be due to the regulation of the transcription factor Dec1 (Human Differentiated Chondrocyte Expressed Gene 1). Such miR-21-5p-mediated regulation of Dec1 has been elegantly described by Kurita et al. utilizing miR-21–5p knock out and Dec1-deficient mice [47].

Another very interesting miRNA in the context of bone healing is miR-125 b. Overexpression of this miRNA is associated with osteoporosis and reduced osteogenic differentiation capacity of MSCs via direct regulation of STAT3 (Signal Transducer and Activator of Transcription 3), p53, and RUNX2, while inhibition of this miRNA promotes mineralization of MSCs in calcium-supplemented media [48], 49].

Additionally, an increasing number of studies have focused on the potential use of miRNAs as biomarkers and predictor of bone healing outcome as specific miRNAs have been reported to be associated with fracture healing phases, patient age, and even with surgical treatment strategies in cases of multitrauma, highlighting their potential to guide clinical decision-making and personalized regenerative approaches [17], 50], 51].

## Tendons and tendon healing

Tendons are another key component of the musculoskeletal system. They are in charge of the stress and force transfer from muscles to bones, allowing for joint movement [52]. Tendons present a hierarchical structure, where collagen molecules assemble into fibrils, which in turn form fibers, fascicles, and finally the tendon itself. Such a structure is stabilized by a dense extracellular matrix, surrounded by the endotenon and epitenon, and maintained by sparsely distributed tenocytes and tenoblasts [52], 53].

Similarly to bone healing, tendon healing undergoes overlapping phases: inflammation, proliferation, and remodeling. However, unlike bones, tendons tend to heal through the deposition of disorganized fibrotic tissue and thus with more scarring. Hence, the complex hierarchical organization of tendons is not often regenerated, and the fibrotic scar that repairs the injured tissue exhibits poor mechanical properties compared to the native tendon, thus increasing the chances of recurrent rupture [54]. For this reason, tendon regeneration remains a major clinical challenge.

**Figure 2:**
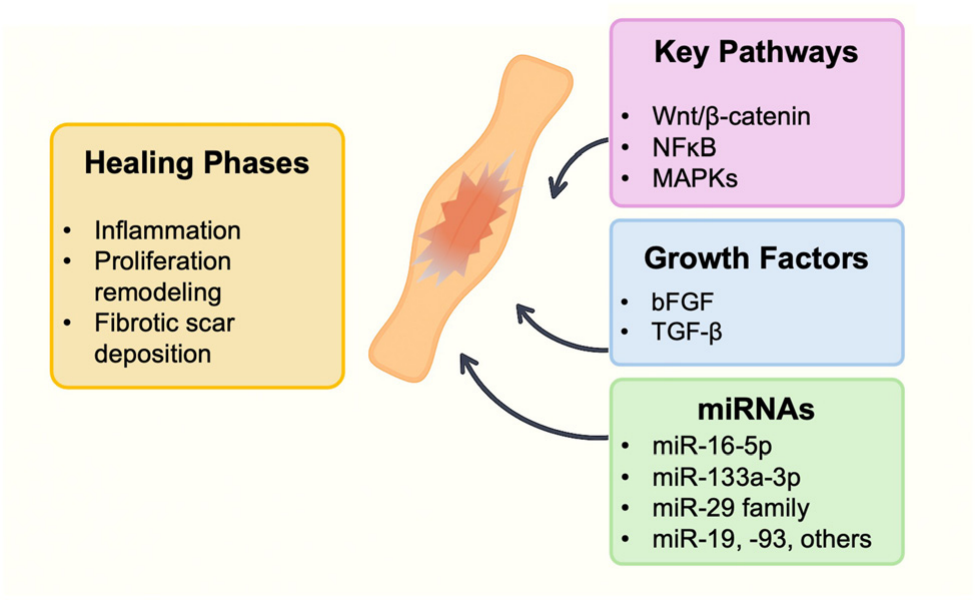
Schematic overview of tendon healing. Following injury, tendons go through overlapping inflammation, proliferation, and remodeling phases. Key signaling pathways (Wnt/β-catenin, NF-κB, MAPKs) modulate tenocyte behavior and matrix remodeling, while growth factors (TGF-β, bFGF) drive extracellular matrix deposition, vascularization, and cell recruitment. Specific microRNAs (e.g. miR-16-5p, miR-133a-3p, the miR-29 family, miR-19/-93) fine-tune fibrotic gene expression and tenogenic differentiation, representing emerging targets to improve regenerative tendon repair.

Most of the efforts aimed to improving tendon regeneration are focused on preventing fibrosis and/or managing fibrotic scar formation. Therefore, pathways implicated in driving this fibrotic phenotype are often the target of tissue engineering strategies for tendon regeneration. This includes the TGF-β pathway, which promotes extracellular matrix deposition and myofibroblast differentiation, and pathways such as the Wnt/β-catenin, NF-κB, and mitogen-activated protein kinases (MAPKs), which are known to be associated with aberrant matrix remodeling, inflammation, and altered tenocyte behavior [55]. In the following section, we will summarize some of the most relevant advances in the use of molecular cues to regulate such pathways and modulate fibrosis in tendon healing (Figure 2).

### Molecular cues in tendon healing

The modulation of signaling pathways involved in tendon development and tendon repair is one of the most promising approaches to manage tendon fibrosis and improve the quality of tendon healing. It has recently been described that the activation of the Wnt/β-catenin signaling pathway suppresses the expression of the tenogenic genes scleraxis, mohawk, and tenomodulin in rat tendon-derived cells. Moreover, the activation of this pathway inhibits the expression of Smad2 and Smad3, key mediators of the TGF-β signaling pathway [56]. Furthermore, it has been reported that inhibiting the Wnt pathway shows reduced tendon inflammation, enhanced tenogenic differentiation, improved load-bearing capabilities, and reduced pain in rodent models of collagenase-induced tendinopathies [57].

Growth factors also play key roles in the regulation of tendon healing and fibrosis. Recent evidence suggests that basic fibroblast growth factor (bFGF) can stimulate the proliferation and differentiation of vascular endothelial cells and promote vascularization during the initial inflammation phase of tendon healing [58]. Delivery of recombinant bFGF, through injection directly into the injured site or by encapsulation in PLGA scaffolds, gelatin hydrogels, or nanoparticles-coated sutures, has been shown to prevent tendon adhesion, improve mechanical strength, and promote better collagen alignment than the untreated controls in studies involving rodent models of tendon injury [59], 60]. It has been described that such positive effects of bFGF in tendon healing are linked to the recruitment of M1 and M2 macrophages, promoting tenocytes proliferation, collagen type I production, and activating NF-κB in tenocytes [58]. Alternatively, contrasting evidence indicates that blocking NF-κB and MAPK signaling pathways alleviates the severity of tendinopathy *in vitro* and *in vivo* [61]. Such a duality is thought to be related to the time-dependent effect of the NF-κB pathway. In the early phases of tendon healing, the activation of NF-κB has an often pro-regenerative role, while the chronic activation can lead to scar formation and inhibition of tenogenic differentiation and overexpression of matrix metalloproteinases (MMPs). This type of duality is also observed in relation to the overactivation of the TGF-β pathways, MAPK pathways, and β-catenin pathways. How to timely regulate such pathways in a regenerative context-dependent manner is still to be elucidated [54].

One promising approach to modulate these crucial pathways to enhance the quality of tendon healing relies on the use of microRNAs. In the context of tendon healing, Peniche Silva et al. reported at least 13 dysregulated miRNAs with potential involvement in fibrosis and scar formation upon an *in vivo* tendon-to-bone enthesis defect. Of these, miR-16-5p and miR-133a-3p were found to be upregulated in the fibrotic portion of the injured tendon [62]. Both of these miRNAs are known to be implicated in the regulation of the TGF-β pathway by inhibition of SMAD2, SMAD3, NOTCH2, TGF-β receptor 1, and other downstream molecules. Thus, these miRNAs have been explored in different applications related to the management of fibrosis in a multitude of organs, although not so much in the context of tendon healing.

The miR-29 family is one of the most frequently investigated in tendon regeneration applications, as it has been demonstrated that the members of this family act as post-transcriptional regulators of collagen and are often dysregulated upon injury and in chronic degenerative tendinopathy [63]. It has been shown that inhibiting miR-29 b-5p, which targets the TGF-β/Smad3 axis, reduces tendon adhesion and controls fibroblast proliferation [64]. Moreover, direct injections of miR-29a, a direct inhibitor of collagen type III, in the site of injury on the digital flexors of horses showed improved tendon healing via reduced expression of collagen type III without affecting levels of expression of collagen type I. Due to the role of collagen type III in the formation of scar tissue, this finding highlighted miR-29a as a master regulator of fibrosis and scar formation. Additionally, levels of circulating miR-25, −19, and −93, which have a potential anti-inflammatory role by modulating the expression of inflammatory cytokines, have been found differentially expressed in patients of chronic tendinopathy compared to healthy controls [65]. Furthermore, miR-29a, −29c, −210–3p, −324–3p, −140–3p, −30a−5p, and −425–5p have been found downregulated, locally and systemically, in chronic tendinopathy and degenerative tendinopathy of the rotator cuff tendon with a progressive decline in the miRNA’s expression depending on the severity of tendon degeneration [65]. Findings such as these pave the way towards the development of novel miRNA-based strategies to improve tendon healing and regeneration.

## Cartilage and cartilage healing

**Figure 3:**
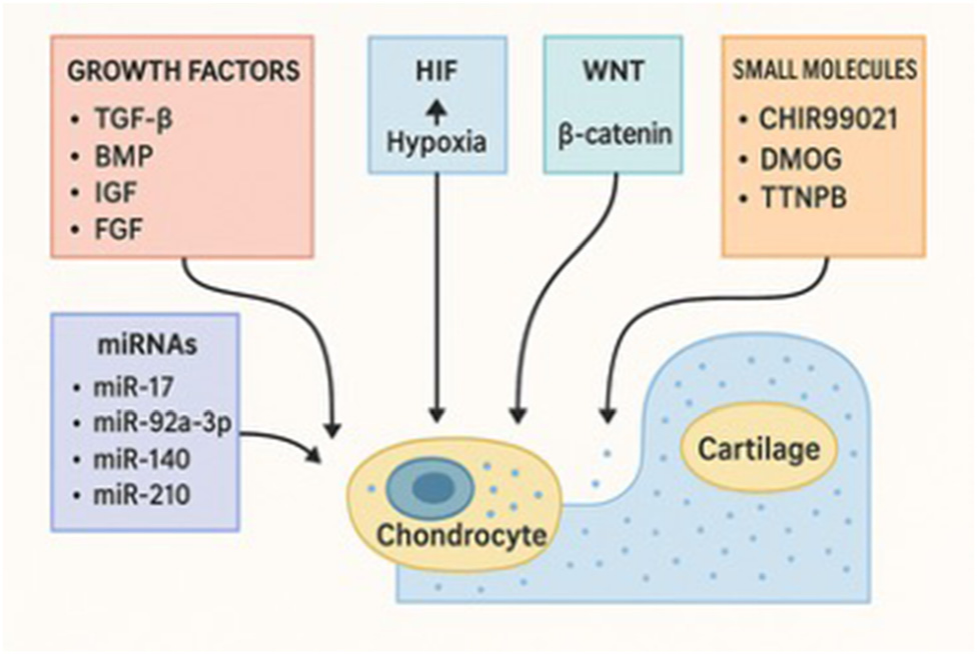
Schematic overview of cartilage healing. Following injury, repair is driven by soluble growth factors (TGF-β, BMP, IGF, FGF) that stimulate Sox9-mediated collagen II and proteoglycan production. Hypoxia-stabilized HIF-1α that enhances matrix gene expression and chondrocyte survival while Wnt/β-catenin signaling balances proliferation and maturation. Small molecules (CHIR99021, DMOG, TTNPB) respectively activating Wnt, sustain HIF-1α, and engage retinoic acid receptors to boost matrix synthesis. Finally, miRNAs (e.g. miR-17, miR-92a-3p, miR-140, miR-210) fine-tune gene networks to restore cartilage structure and function.

Cartilage is a dense, specialized connective tissue composed primarily of chondrocytes embedded within an abundant extracellular matrix (ECM) rich in collagen type II. This provides tensile strength, while proteoglycans attract water and confer resistance to compressive forces [66]. Chondrocytes maintain tissue homeostasis but are sparsely distributed and exhibit low metabolic activity. Unlike other tissues, cartilage is avascular and lacks innervation, which significantly limits its capacity for self-repair [66]. Following injury, cartilage healing is often incomplete or results in the formation of fibrocartilage, which lacks the mechanical properties of native hyaline cartilage. The timeline of cartilage healing varies depending on the extent of injury and the intervention used, but it generally proceeds through distinct phases. Initially, there is an inflammatory response within the first few days, followed by a proliferative phase marked by cell recruitment and matrix synthesis over several weeks. Several key pathways are implicated in this phenomenon [67] (Figure 3).

### Molecular cues in cartilage healing

The TGF-β superfamily plays a pivotal role in cartilage regeneration by influencing chondrocyte proliferation, matrix production, and maintenance of cartilage homeostasis. Among them, TGF-β1 is considered a key anabolic factor for cartilage as well as TGF-β3 [68]. However, it has been shown that TGF-β can also contribute to cartilage degeneration during aging and osteoarthritis (OA), a duality that comes from its ability to activate distinct signaling cascades [67]. Specifically, TGF-β signals through activin-like kinase (ALK)5 and ALK1 receptors, activating Smad2/3 and Smad1/5/8 pathways, respectively. Activation of the ALK5/Smad2/3 axis supports chondrocyte homeostasis, maintaining a quiescent, differentiated phenotype and promoting extracellular matrix production including aggrecan and collagen II, ensuring restoration of cartilage structure and function, while inhibiting hypertrophy [67]. Moreover, activation of the TGF-β/Smad2/3 pathway enhances chondrogenic differentiation of MSCs via modulation of SOX9, collagen II, and aggrecan expression, but also via epigenetic modulation through KDM4B [69], 70]. In contrast, ALK1/Smad1/5/8 axis promotes hypertrophic differentiation via RUNX2, increasing markers such as collagen X, MMP13, and VEGF [71]. Strategies using biomaterial-based delivery of TGF-β have emerged in the recent years. Yang et al. showed that the sustained delivery of TGF-β3 through the use of an optimized 3D-printed functionalized scaffold facilitated the cartilage regeneration in sheep [72]. Another approach using self-assembling peptide hydrogel-based composite scaffold presenting TGF-β1 showed an improved ability of the articular cartilage to regenerate after defect [73]. These studies show that biomaterial-based delivery of TGF-β can create a localized pro-chondrogenic microenvironment, improving the efficacy of tissue-engineered constructs.

BMPs are not only involved in bone regeneration as described before, but they are also important for chondrogenesis and cartilage regeneration. Isoforms such as BMP-2, -4, -6, -7, and -11 are expressed in cartilage. While BMP-2 stimulates chondrocyte proliferation, SOX9 activation, and ECM synthesis through the ALK5/Smad/SOX9 pathway, it can also induce hypertrophy and matrix degradation, particularly via ALK1/Smad/MMP13 [67]. Conversely, BMP-7 supports a stable chondrogenic phenotype, enhances ECM production, and reduces inflammatory signals like IL-1β [67], 74]. Also, BMP-7 showed promising results in maintaining cartilage integrity and reducing inflammation without triggering hypertrophy [75]. Sustained delivery of BMP-2 and BMP-7, such as through the polyhedrin delivery system, has shown better cartilage repair and integration in murine models compared to soluble forms [76]. In an OA defect, the combination of TGF-β3 and BMP-7 showed a higher chondrocyte redifferentiation than the separated treatments in both normoxia and hypoxia, showing the importance of finding a good balance [77]. Thus, the potential of TGF-β and BMPs in cartilage regeneration lies in the precisely controlled signaling to favor chondrogenic fate over osteogenic differentiation.

Insulin growth factor-1 (IGF-1) plays a central anabolic role in cartilage metabolism by stimulating ECM synthesis and maintaining the chondrocyte phenotype. It promotes matrix production more than proliferation and helps prevent progression to hypertrophy. IGF-1 acts synergistically with TGF-β1 and boosts its chondrogenic effects. IGF-1 is able to activate PI3K/Akt and MEK/ERK pathways, leading to chondrogenesis, ECM synthesis, and inhibition of catabolic processes [78]. In 2021, Maihöfer et al. demonstrated that sustained IGF-1 overexpression, via biomaterial-guided rAAV (recombinant adeno-associated virus) gene transfer, significantly improved full-thickness cartilage repair in minipigs, enhancing matrix deposition and reducing peri-defect inflammation over one year [79]. Moreover, hydrogels delivering both IGF-1 and MSCs were shown to accelerate chondrogenic differentiation and glycosaminoglycan (GAG) deposition in rabbits [80].

Fibroblast growth factors, particularly FGF-2 and FGF-18, are key regulators of cartilage homeostasis [67]. FGF-2 has shown both regenerative and catabolic effects. It supports cartilage repair by expanding chondroprogenitors and maintaining ECM through PI3K/Akt signaling and can act together with BMPs to enhance chondrogenesis. However, in human chondrocytes, FGF-2 has been associated with upregulation of catabolic enzymes, such as MMP-13, ADAMTS-4/-5, suppression of ECM synthesis, and increased expression of pro-inflammatory cytokines [67]. In contrast, FGF-18 consistently exhibits anabolic effects. Acting via FGFR3, it promotes chondrocyte proliferation and ECM synthesis, leading to increased cartilage thickness in both porcine and human models [81]. Recombinant human FGF-18 (rhFGF-18) has shown to improve cartilage quality and collagen II expression 24 weeks after microfracture in horses [81].

The Wnt signaling plays a dualistic, context-dependent role in cartilage regeneration, where precise regulation promotes repair, while excessive activation drives degeneration [82]. Regulated canonical Wnt/β-catenin signaling supports chondrocyte proliferation, matrix production, and progenitor cell activation during cartilage repair, highlighting the need for signaling balance [67]. In contrast, in OA, Wnt hyperactivation leads to IGF-1 upregulation and downstream catabolic responses, including elevated MMP-13 expression and cartilage degradation; notably, cartilage-specific deletion of *Igf1* confers protection against joint damage [83]. Additionally, hypoxic stabilization of ANP32A through hypoxia-inducible factors (HIFs) has been shown to suppress Wnt hyperactivation and preserve cartilage integrity in osteoarthritis [82].

HIFs, particularly HIF-1α and HIF-2α, are important modulators of cartilage regeneration, modulating cell survival, metabolic reprogramming, and ECM synthesis under low oxygen conditions. HIF-1α primarily promotes anabolic processes by enhancing the expression of chondrogenic genes such as *SOX9*, *COL2A1*, and *ACAN*, while also shifting cell metabolism toward glycolysis to support cell viability in avascular cartilage environments [84], 85]. HIF-1α also suppresses the catabolic enzymes MMP-13 and ADAMTS5 to support chondrogenic differentiation of MSCs. In contrast, HIF-2α has been associated with both physiological and pathological roles, supporting cartilage development under controlled conditions, but also driving the expression of catabolic enzymes like *MMP13* and *ADAMTS5* in OA progression [86].

Small molecules offer precise modulation of signaling pathways crucial for cartilage regeneration. CHIR99021 increases the chondrogenic capacity of hMSCs through modulation of the Wnt pathway (inhibition of GSK3β) [87]. Dimethyloxallyl-glycine is able to mimic hypoxia by stabilizing HIF-1α, leading to region-specific increases in collagen type II deposition; however, it does not necessarily improve overall matrix production [88]. TTNPB, a retinoic acid analog, has similarly shown capacity to drive early chondrogenic specification when used alongside BMP-2 in stem cell‐derived constructs [89]. Association of TD-198946 with BMP-2 has also been shown to enhance chondrogenic differentiation after cartilage lesion, highlighting the potential of combined treatments [90].

MiRNAs and long non-coding RNAs (lncRNAs), are critical post-transcriptional regulators of cartilage regeneration. They modulate the earlier highlighted key signaling pathways such as Wnt, HIF, TGF-β/BMP, PI3K/Akt, and indirectly, IGF and FGF, influencing chondrocyte behavior, ECM turnover, and cartilage homeostasis. Among these, miR-140-5p/3p is cartilage-specific and maintains ECM integrity by targeting MMP-13 and ADAMTS5, promoting SOX9 expression, and modulating Wnt and TGF-β signaling [91]. By targeting HIF, miR-210 promotes chondrogenic differentiation of bone marrow MSCs by increasing COL2A1 and SOX9, while suppressing adipogenic differentiation [92]. Another multifunctional regulator, miR-17, a member of the miR-17-92 cluster, supports cartilage homeostasis by targeting MMP-3, MMP-13, ADAMTS5, and HIF-1α, reducing catabolic activity. miR-17 also interacts with TGF-β/BMP pathways through competing endogenous RNA (ceRNA) networks, promoting SOX9-mediated chondrogenesis and limiting hypertrophy [93]. In parallel, lncRNAs also play essential roles. LncZFHX2, upregulated under hypoxia via HIF-1α, forms a transcriptional complex with KLF4 to activate the DNA repair protein RIF1, delaying chondrocyte senescence and preserving matrix integrity. Its deletion accelerates cartilage degeneration, underscoring its protective function [94].

## Skeletal muscle and molecular cues in muscle healing

**Figure 4:**
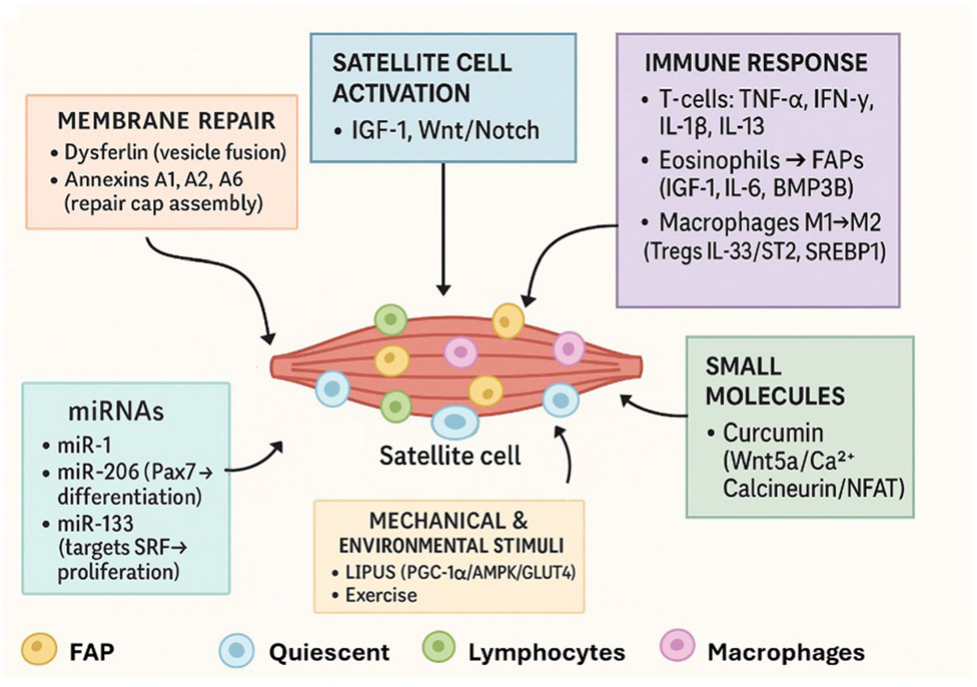
Schematic overview of skeletal muscle regeneration. Following injury, membrane repair proteins (dysferlin, annexins A1/A2/A6) reseal the sarcolemma, while satellite cells are activated by IGF-1 and Wnt/Notch signaling. The immune response, T-cell TNF-α/IFN-γ/IL-18/IL-13, eosinophil-driven FAP IGF-1/IL-6/BMP3B, and macrophage M1→M2 transition with Treg IL-33/ST2/SREBP1, further modulates repair. Moreover, mechanical and environmental stimuli enhance myogenesis; specific miRNAs (miR-1, miR-206, or miR-133) adjust satellite cell behavior. Small molecules like curcumin (via Wnt5a/Ca^2+^/calcineurin–NFAT) boost myogenic gene expression.

Skeletal muscle enables voluntary movement and posture, regulates energy balance by storing and releasing nutrients, and produces heat during contraction to help maintain body temperature. These muscles are made of long, cylindrical, multinucleated fibers bundled into fascicles. Each fiber contains repeating sarcomeres, composed of actin and myosin filaments, giving skeletal muscle its characteristic striated appearance [95]. After injury, skeletal muscle regeneration is a highly coordinated process involving membrane repair, inflammatory resolution, metabolic reprogramming, and structural restoration [96] (Figure 4).

Following skeletal muscle injury, rapid stabilization of the sarcolemma is essential to prevent fiber necrosis and initiate effective regeneration. Key components of the membrane repair machinery include dysferlin, which mediates vesicle fusion at the damage site, and annexins (A1, A2, A6), which assemble into repair caps in a calcium-dependent manner to seal membrane breaches [96].

After stabilization, muscle satellite cells (MuSCs) will be stimulated to trigger muscle regeneration. Located beneath the basal lamina in a quiescent state, these cells are activated in response to injury through signals such as IGF-1, and Wnt/Notch pathway cues [97]. MuSCs upregulate glycolysis and increase mitochondrial biogenesis [98], 99]. These processes are facilitated by a variety of signals, such as the transcription factor Yin Yang 1 which is able to control both glycolytic and mitochondrial gene expression in MuSCs [100]. Macrophage-derived glutamine also supports proliferation and differentiation of MuSCs through activation of mTOR [101]. Altogether, this leads to new myocytes but also renewal of the stem cell niche. Once activated, MuSCs proliferate and commit to the myogenic lineage via transcription factors including MyoD and Myf5, ultimately differentiating to myocytes and fusing to repair or replace damaged myofibers [96], 102].

His is concomitant with infiltration of immune cells, starting with T-cells, recruited by chemoattractants produced by resident macrophages and releasing cytokines including TNF-α, IFN-γ, IL-1β, and IL-13, triggering the aforementioned metabolic pathways. Furthermore, eosinophils are also present and stimulate fibro/adipogenic progenitors (FAPs) via IL-4 and IL-13. FAPs are stromal cells that dynamically interact with MuSCs [97]. FAPs proliferate and clean the necrotic debris thanks to the implication of several molecular pathways including IL-6, promoting macrophage migration and myoblast proliferation, IGF-1, promoting MuSCs activation and differentiation, or BMP-3B/GDF-10 [97], 103]. Additionally, FAPs increase WNT1-inducible-signaling pathway protein 1, promoting expansion and myogenic commitment of MuSCs. Reciprocally, EGF secreted by MuSCs promotes FAP activation and differentiation into myofibroblasts. Finally, FAP-derived myofibroblasts increase ECM production to serve as support for new myofibers [104], 105].

Injury also induces a wave of macrophage infiltration, initially dominated by pro-inflammatory M1 macrophages, which later transition to an anti-inflammatory M2 phenotype [97]. This switch is facilitated by regulatory T cells (Tregs) via IL-33/ST2 signaling and amphiregulin secretion, ultimately fostering a pro-regenerative environment [106]. Additionally, macrophage-derived SREBP1 has been implicated in modulating the inflammatory environment post-injury, promoting a pro-regenerative niche within damaged muscle [107]. As repair progresses, pro-inflammatory macrophages promote FAP apoptosis through TNF signaling, clearing FAP excess and preventing fibrosis [105].

Mechanical and environmental stimuli also modulate myofiber regeneration. Low-intensity pulsed ultrasound enhances satellite cell and myoblast activity by activating PGC-1α/AMPK/GLUT4 signaling, improving mitochondrial function and glucose uptake [108]. Similarly, acute and long-term swimming exercise transiently activates inflammatory and oxidative stress pathways, which can contribute to both repair stimulation and muscle damage, depending on intensity and duration [109].

Compounds as curcumin further influence myofiber regeneration through activation of the non-canonical Wnt5a/Ca^2+^/calcineurin/NFAT signaling, enhancing calcium influx and promoting myoblast differentiation [110]. Regarding ECM deposition, while TNF-α is shown to be able to reduce fibrosis by increasing FAP clearance, TGF-β1 is doing the opposite. Lemos et al. showed that treatment with Nitolinib blocks the effect of TGF-β1 and reduces muscle fibrosis [105].

As for other tissues, miRNAs are playing an important role in muscle regeneration. In this review, we focused on three miRNAs well-known in muscular tissue; miR-1 and miR-206. These miRNAs are upregulated in activated satellite cells and induce a decrease of Pax7 protein levels, reducing proliferation and favoring myogenic differentiation [111]. In contrast, miR-133, which is co-transcribed with miR-1, targets serum response factor (SRF) to restrain differentiation and support myoblast proliferation during the early phases of regeneration [112]. Interestingly, the same study showed that miR-1 can also enhance myogenic differentiation by targeting histone deacetylase 4 (HDAC4), relieving the repression of muscle‐specific gene expression [112]. Finally, during muscle repair, miRNAs also participated in communication between cells. Activated FAPs secrete extracellular vesicles (EV) enriched in miR-127-3p, targeting specific genes in MuSCs to promote their myogenic differentiation. Conversely, MuSCs release EVs containing miR-206 instructing FAPs to suppress their adipogenic program [113].

## Conclusions

The tissues in the musculoskeletal system possess a remarkable yet limited capacity for self-repair, especially when faced with complex injuries or chronic degeneration. This review highlighted the most investigated molecular cues with the potential to guide and enhance the regenerative process after injury in the context of bone, tendon, cartilage and muscle. Molecular cues such as growth factors, non-coding RNAs, and ECM components, rather than acting in isolation, form dynamic and interconnected signaling networks. Thus, decoding and leveraging these networks offers the potential to move beyond symptomatic treatments toward true tissue regeneration. Moreover, by understanding how specific signals influence inflammation, cell recruitment, differentiation, matrix deposition, and remodeling, it is possible to design more precise and context-specific interventions and aid process of repair and healing. However, while much has been achieved, further research is needed to translate these advances into safe, effective, and accessible clinical therapies.
